# Is Competition the Default Configuration of Cross‐Sensory Interactions?

**DOI:** 10.1111/ejn.70233

**Published:** 2025-08-26

**Authors:** Melissa Monti, Sophie Molholm, John J. Foxe, Cristiano Cuppini

**Affiliations:** ^1^ Department of Electrical, Electronic, and Information Engineering Guglielmo Marconi University of Bologna Bologna Italy; ^2^ The Cognitive Neurophysiology Laboratory, Departments of Pediatrics and Neuroscience Albert Einstein College of Medicine Bronx New York USA; ^3^ The Frederick J. and Marion A. Schindler Cognitive Neurophysiology Laboratory, The Del Monte Institute for Neuroscience, Department of Neuroscience University of Rochester School of Medicine and Dentistry Rochester New York USA

**Keywords:** autism spectrum disorder, multisensory integration, neurocomputational modelling, switch cost, typical development

## Abstract

Several theories have been proposed about the default configuration of the brain's networks underlying unisensory and multisensory processing abilities and the development of multisensory integration during childhood. Recent empirical findings from animal models and behavioral data collected from typically developing (TD) children and children with autism spectrum disorder (ASD), however, are consistent with the idea that in the immature brain, prior to systematic cross‐sensory exposures typically encountered in everyday life, the individual sensory systems interact in a competitive manner. Which neural architecture and mechanisms best describe the brain's naïve configuration are still unknown. To fill this gap, this study investigates how sensory modalities interact in the young brain by comparing the predictions of two alternative biologically plausible neuro‐computational models to empirical data. The neural substrates responsible for the altered development of multisensory integrative processes observed in ASD children are also investigated. Linking the framework suggested by empirical data to a plausible neural implementation, our results challenge the classical notion of cross‐sensory brain organization at birth, whereby the various sensory pathways do not initially interact. Instead, we suggest that direct inhibitory interactions between sensory modalities are taking place in the immature brain, and we suggest that these inhibitory interactions play a crucial role in the altered multisensory perceptual abilities of children with autism.

## Graphical abstract

Neurocomputational modelling suggests that early cross‐sensory interactions are inherently competitive. Replicating ASD behavioral data requires stronger cross‐sensory competition, consistent with the hypothesis of delayed maturation.

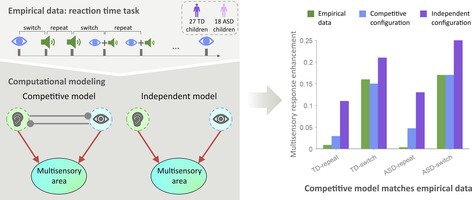

**Abbreviations**
ASD: autism spectrum disorders
ISI: inter‐stimulus‐interval
MRE: multisensory response enhancement
MSI: multisensory integration
RT: reaction time
TD: typically developing
TWI: temporal window of integration

## 1 Introduction

The intricate symphony of human behaviors, movements, executive functions, language, and social interactions relies on intact perceptual functioning (Foxe et al. 2015; Kolb 1984; Pinker 2010). Central to the orchestration of the intricate spectrum of these functions is multisensory integration (MSI), the ability of the neural architecture to integrate sensory information from different modalities. This capacity enables us to navigate and make sense of our environment, influencing not only basic sensory processing but also higher order cognitive functions, thereby shaping our interactions with both the social and physical world.

Given its essential role in such a wide array of brain functions, understanding the neural mechanisms underlying MSI is key to comprehending brain function. This includes examining how integrative mechanisms are in their default configuration at birth and how integrative abilities are acquired across development and the lifespan.

Experimental findings in both humans and animals show that multisensory integration is immature at birth and follows a protracted developmental trajectory, often extending into adolescence (Brandwein et al. 2011; Burr and Gori 2012; Crosse et al. 2022; Lewkowicz and Ghazanfar 2009; Ross et al. 2007, 2011). Moreover, the development of such processes depends crucially on the specific sensory experience with cross‐modal cues (Bahrick and Lickliter 2000; Carriere et al. 2007; Rowland et al. 2014; Wallace 2004; Wallace and Stein 1997; Xu et al. 2014; Yu et al. 2010). For example, studies that have systematically manipulated or compromised early sensory cues (e.g., by rearing animals in darkness or omnidirectional masking sound) found a correlation between the emergence of integrative abilities and the specific sensory experience perceived during the maturation process (Carriere et al. 2007; Wallace 2004; Xu et al. 2014; Yu et al. 2010). This is likely due to the fact that optimal integration relies on extensive engagement with, and is shaped by, the sensory environment, as well as sensory‐driven developmental changes in neural architecture and connectivity (e.g., Merabet and Pascual‐Leone 2010).

While significant progress has been made in understanding how the environment shapes the development of MSI abilities, much less is known about how changes in the brain's architecture contribute to the acquisition of these abilities. To address this fundamental question, the present work will extend computational frameworks of MSI that we have developed to test possible neural mechanisms (1) characterizing the immature architecture of multisensory processing, (2) how this differs from the mature configuration, and (3) how it differs in ASD compared to typically developing (TD) children.

Seminal work on MSI has illustrated that for neural integration or behavioral facilitation to occur, the constituents of a cross‐sensory stimulus must onset within a narrow temporal window—approximately 200 ms—though this window can vary for different stimuli (e.g., briefer windows for simple punctate stimuli and longer windows for complex stimuli such as audiovisual speech) (Diederich and Colonius 2015; Mégevand et al. 2013; Meredith et al. 1987; Miller et al. 2015; Stein and Meredith 1993; Stevenson et al. 2012; Stevenson and Wallace 2013; van Eijk et al. 2008; Vatakis and Spence 2006). Cross‐sensory switch effects occur when the interval between sensory components exceeds this TWI, with behavioral costs for trials preceded by a stimulus in a different sensory modality versus trials in which the sensory modality repeated (Barutchu and Spence 2021; Crosse et al. 2022; Shaw et al. 2020; Spence et al. 2001; Vanneau et al. 2025).

Recently, Crosse et al. (2022) examined the development of MSI and cross‐sensory switch costs in children using a simple reaction time (RT) task with stimuli presented in random order and varying stimulus onset asynchronies between 1 and 3 s. They found that, in younger children (~6 years old), consecutive stimuli from different sensory modalities interacted competitively. Moreover, they described the maturation of multisensory interactions in children for temporally aligned multisensory stimuli, specifically identifying a state of competition between sensory modalities in younger children (~6 years of age), which turns to facilitation over the course of development.

This finding aligns with Yu et al. (2019) animal model: Using auditory and visual stimuli to test the integrative abilities of multisensory neurons in adult cats raised in the dark, the authors found that spatiotemporally concordant audiovisual stimuli failed to evoke enhanced neural responses and instead led to response suppression. In this case, integrative responses only appeared after extended experience with congruent multisensory AV stimuli. Although care is needed in interpreting these findings in a developmental context (since they were acquired from the mature brain), they suggest that interactions across the sensory systems initially involve competition, which later shifts to facilitation with increased multisensory experience (typically during early development).

Moreover, Crosse et al. (2022) showed that such transition is delayed in children with autism spectrum disorders (ASD). This finding is in agreement with a wide cohort of previous scientific studies, demonstrating that multisensory processing is often impaired in ASD individuals, with improvements in their ability to integrate sensory information occurring more slowly compared to their TD peers (Beker et al. 2018; Brandwein et al. 2013; Cuppini et al. 2017; Foss‐Feig et al. 2010; Foxe et al. 2015; Stevenson et al. 2012; Stevenson, Segers, et al. 2014; Stevenson, Siemann, Schneider, et al. 2014; Stevenson, Siemann, Woynaroski, et al. 2014; Taylor et al. 2010; Wallace and Stevenson 2014). Importantly, these studies also suggested that these impairments are related to atypical neural development of multisensory processing mechanisms (Foxe et al. 2015), which may arise from atypical sensory experiences with cross‐modal cues (Cuppini et al. 2017).

Despite extensive research into the development of MSI, much remains unknown about the neural mechanisms underlying the default competitive configuration of multisensory processing, how the brain handles stimuli at varying temporal discrepancies, and how experience shapes the neural architecture involved. In our neurocomputational model (Cuppini et al. 2020), we identified plausible neural mechanisms that influence the TWI, providing insight into cross‐modal interactions in the mature brain. In this study, we extend this computational framework to explore (1) the mechanisms characterizing the default architecture of sensory processing, (2) how these mechanisms differ between children and adults, and (3) how children with ASD differ from their TD peers.

## 2 Materials and Methods

Here, to test possible neural mechanisms characterizing multisensory interactions in young children and how these change over development, we used neurocomputational modelling to compare two distinct synaptic architectures simulating children's behavior based on data reported in Crosse et al. (2022). The two model architectures and the implemented mechanisms are described qualitatively. The formal mathematical description of the model, including all equations, is provided in the Supporting Information together with criteria for parameter assignment and parameter values (see Table S1).

Unless otherwise specified, throughout the manuscript, we use the terms *competition* and *facilitation* to refer to behavioral outcomes (i.e., changes in RTs), and not to neural responses. This is consistent with the scale of our model, which operates at the mesoscale, where each model unit represents the activity of an entire ensemble of neurons and where simulated outputs are intended to reflect behavioral performance rather than single‐unit neural responses.

### 2.1 The Neuro‐Computational Model

#### 2.1.1 Architecture

The original model (Cuppini et al. 2020) was developed to simulate the behavioral task implemented by Crosse et al. (2022), which builds upon the seminal work of Molholm et al. (2002). This model focuses on multisensory interactions in the time domain: Subjects were required to respond as fast as possible to auditory and visual stimuli, alone or combined, presented in a random sequence, with an inter‐stimulus‐interval (ISI) varying between 1 and 3 s. Given the experimental set‐up of Crosse et al. (2022), we did not require either multiple units sensitive to different spatial positions in each sensory area, as implemented in our previous neurocomputational models (Cuppini et al. 2012, 2014, 2017, 2018; Cuppini, Magosso, et al. 2011; Cuppini, Stein, et al. 2011; Magosso et al. 2012; Ursino, Crisafulli, et al. 2017; Ursino, Cuppini, et al. 2017; Ursino et al. 2019) nor did we need multiple sensory regions sensitive to different input features, as necessary to realize semantic memory models (Cuppini et al. 2009; Ursino et al. 2009, 2010, 2011, 2018). Therefore, in the model, each region was simulated with a single neural element for simplicity.

**FIGURE 1:**
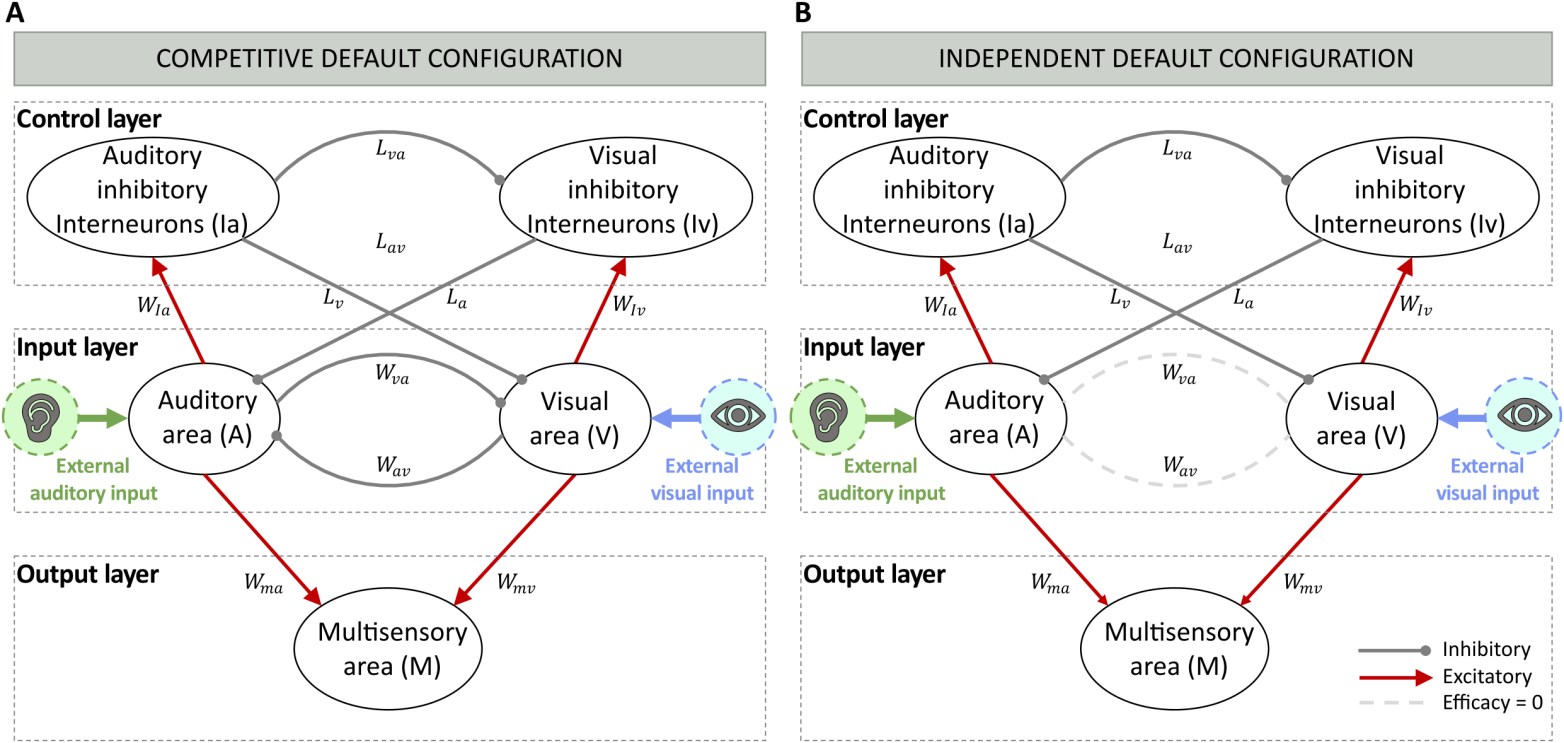
Two alternative model's structures. (A) The competitive default configuration (simulating subjects of 6–7 years of age). A and V represent the auditory and visual regions, responsible for the first sensory processing, and implementing the input layer. They exchange direct inhibitory synapses, implementing a cross‐modal competition. M is the multisensory output region. It is responsible for generating simulated reaction times (RTs) to the external stimuli. I_a and I_v are unimodal inhibitory areas, excited by the input layer and implementing a control mechanism via feedback inhibitory synapses and a winner‐takes‐all dynamics. (B) The independent default configuration. Its synaptic architecture differs for the absence of cross‐modal synapses (W_av, W_va), so the model does not have direct interaction among sensory modalities. Red lines represent excitatory connections; blue lines represent inhibitory synapses.

The model structure consists of three layers (Figure 1). The first layer, called the “input layer,” represents the primary unisensory auditory and visual areas (A and V in Figure 1), which are activated by external stimuli of the corresponding sensory modalities ($I_{0}^{a}$ and $I_{0}^{v}$, respectively) and provide the first sensory processing step in this simplified model. The onset, duration, and presentation rate (ISI) of the stimuli were chosen to mimic the experimental setup of Crosse et al., whose data are compared to our model results. Specifically, external stimuli are excitatory inputs with an assigned efficacy, and a duration of 60 ms, presented at a mean rate of one every 2 s (the mean ISI in Crosse et al.'s experiment), with an ISI ranging from 1 to 3 s.

The “control layer” ($I_{a}$ and $I_{v}$ in Figure 1) implements a cross‐sensory competition between the two sensory modalities when stimuli are presented sequentially: modality‐specific inhibitory interneurons produce a long‐lasting inhibition of the input region processing external inputs of the other sensory modality. It is worth noting that, since the dynamics of this mechanism are slow, the processing of the current stimulus is affected by the previous one, when of the opposite sensory modality.

Finally, the third layer, a multisensory output area (M in Figure 1), is used to mimic the behavioral responses (RTs) of subjects to external stimuli: the elicited activity in the output region is compared with a fixed threshold, φ (10% of the maximum neurons' activity), to evaluate the simulated RTs, computed as the time interval between the instant of input presentation and the instant when the evoked activity in the output area reaches the threshold.

#### 2.1.2 Connectivity and Implemented Mechanisms

The two input regions send long‐range excitatory projections, $W_{\mathrm{ma}}$ and $W_{\mathrm{mv}}$, to the read‐out multisensory layer (M region), characterized by fast dynamics.

Additionally, neural elements belonging to the input layer send excitatory synapses ($W_{\mathrm{Ia}}$ and $W_{\mathrm{Iv}}$) towards the modality specific inhibitory areas ($I_{a}$ and $I_{v}$) of the control layer. This layer implements a winner‐takes‐all mechanism by means of reciprocal inhibitory synapses, $L_{\mathrm{av}}$ and $L_{\mathrm{va}}$, and the “winning” sensory modality exerts an inhibitory effect on the incoming stimulus of the opposite modality (if present), through feedback inhibitory synapses, $L_{a}$ and $L_{v}$. These synaptic connections are characterized by a slow dynamic, thus resulting in a long‐lasting inhibitory effect, mediating a cross‐sensory competition characterized by a long‐lasting slower dynamic, imposing its inhibitory effect on the following input.

Model parameters were manually tuned to qualitatively reproduce the key patterns observed in the empirical data reported by Crosse et al. (2022) for the 6‐ to 7‐year‐old group. In order to balance biological plausibility and empirical validity, parameter tuning was guided by constraints from the literature (e.g., known sensory latencies and activation dynamics), as well as prior modelling studies using similar architectures (Cuppini et al. 2012, 2018; Cuppini, Magosso, et al. 2011; Magosso et al. 2012; Ursino, Cuppini, et al. 2017). To ensure that the final parameter set did not simply overfit one specific dataset or configuration, we validated the model on additional experimental paradigms (as detailed in Section 2.2). In particular, we tested the model's ability to account for behavioral effects under different ISI values and across inter‐individual variability, demonstrating that it could robustly capture the modulation of modality switch costs without further tuning.

#### 2.1.3 Alternative Structures

As stated above, to identify the mechanisms that may characterize an initial state of sensory processing, one that is subsequently altered based on systematic multisensory experiences, we tested two alternative hypotheses. In the first case, the “Independent default configuration” (Figure 1B), the model provides independent processes for the two sensory modalities. The underlying architecture is the one described above, where the sensory modalities interact through the inhibitory feedback, characterized by a slow temporal dynamic, and at the level of the output region, M, receiving the feedforward projections from the unisensory input regions.

The second implementation, the “Competitive default configuration” (Figure 1A), instead, presents the same synaptic structure, but in addition, the auditory and visual areas are reciprocally connected via cross‐modal inhibitory connections, $W_{\mathrm{av}}$ and $W_{\mathrm{va}}$, characterized by fast dynamics. With such a structure, in the case of a multisensory stimulus, the input regions mutually suppress one another, resulting in a fast competition between the two sensory modalities.

### 2.2 Assessment of Network Performance

To discriminate between the two alternative architectures of the network and decide which one is the more suitable to be implemented in the model to simulate the default configuration and to best reproduce and explain the empirical data, we performed an analysis of the results obtained by Crosse et al. (2022). More specifically, we analyzed the case of Repeat and Switch conditions, for unisensory and multisensory stimuli, only for the subpopulation of children of 6–7 years of age, in TD and ASD populations. We focused on this age group because it was the youngest analyzed by the researchers, so that it is the closest to the default configuration of the brain's circuits. Then we compared these results with the simulated results obtained with both models.

To test and compare the network's behavior in the two possible configurations and identify the most likely structure, we ran several simulations, presenting sequences of unisensory (auditory‐alone and visual‐alone) and multisensory stimuli (audiovisual inputs) with an ISI of 2000 ms (the mean value of those utilized in Crosse's paradigm). Simulated RTs were computed as the time interval between the instant of input presentation and the instant when the evoked activity in the output area reached the threshold. These results were analyzed separately based on the respective input modality. Furthermore, we discriminated between “Repeat” trials (the preceding stimulus belonged to the same sensory modality) and “Switch” trials (the preceding stimulus was of a different sensory modality). The inputs used in each repetition, for each stimulus condition, were randomly chosen from a uniform distribution in order to replicate the within‐subject variability of sensory stimuli in a real environment. This has been obtained by adding a noisy component to the mean value of the auditory and visual stimuli, generated from a uniform distribution with zero mean and maximum and minimum bounds equal to ± an assigned percentage of the basal value (i.e., I = I_0_ ±𝑛𝑜𝑖𝑠𝑒%·I_0_, with 𝑛𝑜𝑖𝑠𝑒% as high as the 20% in the simulations). Therefore, for every stimulus configuration, first, we computed the mean RTs, obtained from 100 simulations with the same input condition, then, we compared these model's RTs with the RTs from the 6‐ to 7‐years‐of‐age population in Crosse et al. (2022).

To quantitatively assess which of the two network architectures better accounts for the empirical data, we computed the multisensory response enhancement (MRE) for both the experimental and simulated RTs. MRE provides a normalized index of intersensory facilitation (or inhibition) and is conceptually analogous to neurophysiological measures of multisensory enhancement at the neuronal level—typically defined as the increase in a neuron's response to a multisensory stimulus relative to the response to the most effective unisensory input (Anastasio et al. 2000; Diederich and Colonius 2004; Meredith and Stein 1986). MRE was computed as

(1) $$\mathrm{MRE}=\frac{\min\left({\bar{\mathrm{RT}}}_{A,V}\right)-{\bar{\mathrm{RT}}}_{\mathrm{AV}}}{\min\left({\bar{\mathrm{RT}}}_{A,V}\right)}\times 100$$

where ${\bar{\mathrm{RT}}}_{A}$, ${\bar{\mathrm{RT}}}_{V}$, and ${\bar{\mathrm{RT}}}_{\mathrm{AV}}$ refer to the observed mean RTs to auditory, visual, and audiovisual stimuli, respectively. The term $\min\left({\bar{\mathrm{RT}}}_{A,V}\right)$ thus represents the fastest unisensory mean RT. MRE was computed separately for repeat and switch conditions for both ASD and TD, using the corresponding mean RTs for each condition and for each population.

### 2.3 Model Validation

A neurocomputational model is useful to the extent that it bridges and explains several phenomena. Simply stated, to be utilized to generate testable predictions and suggest new experiments, the model needs to be validated. For this reason, once we identified the more plausible neural architecture to explain the default configuration of sensory interactions, we validated our network by (i) manipulating the ISI and (ii) introducing inter‐subject variability. These manipulations allowed us to simulate changes in the experimental setup and account for individual differences among participants, respectively. To assess whether the model could generalize to these new conditions without further parameter tuning, we compared its predicted RTs to the empirical RTs reported by Crosse et al. (2022).

We examined the effect of ISI by varying it randomly between 1 and 3 s, the two extremes used in the original experiment by Crosse and colleagues. In a first set of simulations, referred to as the “Short ISI” condition, we sampled ISIs from a uniform distribution ranging from 1 to 1.5 s, thus presenting the model with stimuli occurring closer in time than the average ISI. In a second set, the “Long ISI” condition, ISIs were sampled from 2.5 to 3 s, presenting the model with stimuli spaced further apart in time than the mean ISI. The duration of the ISIs was modified in the case of Repeat and Switch conditions for both unisensory and multisensory stimuli, and the predicted RTs were compared with the empirical RTs.

To simulate inter‐participant variability, that is, the behavioral differences among the individuals involved in the Crosse experiment, we introduced a further noise component to the parameters describing the different architectural mechanisms implemented in the model. Specifically, a noisy component was introduced on the feedforward synapses. To do so, the effectiveness of these synapses was generated from a uniform distribution on open interval from zero to a maximum value, higher than the basal synaptic strength of these connections (for TD participants: $W_{\mathrm{ma}}=$ [0–6], $W_{\mathrm{mv}}=$ [0–16]; for ASD: $W_{\mathrm{ma}}=$ [0–3], $W_{\mathrm{mv}}=$ [0–8]). Each value of this parameter can be considered representative of a different simulated individual. Similarly, a change in the effectiveness of inhibitory feedback synapses was realized by adding a random component to their basal value. In this case, the noisy component was generated from a uniform distribution with zero mean and maximum and minimum bounds equal to ± an assigned percentage of the basal value (i.e., 𝐿 = ±𝑛𝑜𝑖𝑠𝑒%·𝐿_0_, with 𝑛𝑜𝑖𝑠𝑒% = 50%). Each value of this parameter can be considered representative of a different simulated participant. As described before, for each simulated participant, we evaluated mean RTs for every stimulus configuration (unisensory/multisensory and repeat/switch conditions), over 100 presentations.

### 2.4 ASD Simulations

As discussed above, ASD subjects integrative abilities mature later in life. Since one of our aims was to estimate which architectural mechanism differs, and how it differs, in this population, we ran several simulations, testing the network in its default configuration, selectively modifying different parameters. For each parameter's configuration, we replicated the same simulations described above, analyzing the behavior of the model for unisensory and multisensory stimuli, in the Repeat and Switch conditions, and then we compared these results with the youngest ASD children's data. Once we defined the new set of parameters simulating the ASD population, we analyzed the differences between TD and ASD simulated conditions to identify the altered mechanisms in the latter.

## 3 Results

In the following section, we present the results we obtained from these simulations. We have structured this section in a way that reflects its sequential nature, detailing each step we carried out, and elucidating how each step influenced the subsequent one. Overall, our findings shed light on (i) the synaptic arrangement most likely to implement the default configuration suggested by the empirical results; (ii) how this configuration differs from that observed in adults; and (iii) how children with ASD differ from their TD peers. In addition, validating the model's ability to generalize across conditions allowed us to explore how it handles temporal disparities between auditory and visual inputs and which mechanisms may account for observed inter‐individual variability in behavioral responses.

### 3.1 Data Analysis From Crosse et al.'s Experiment (2022)

**FIGURE 2:**
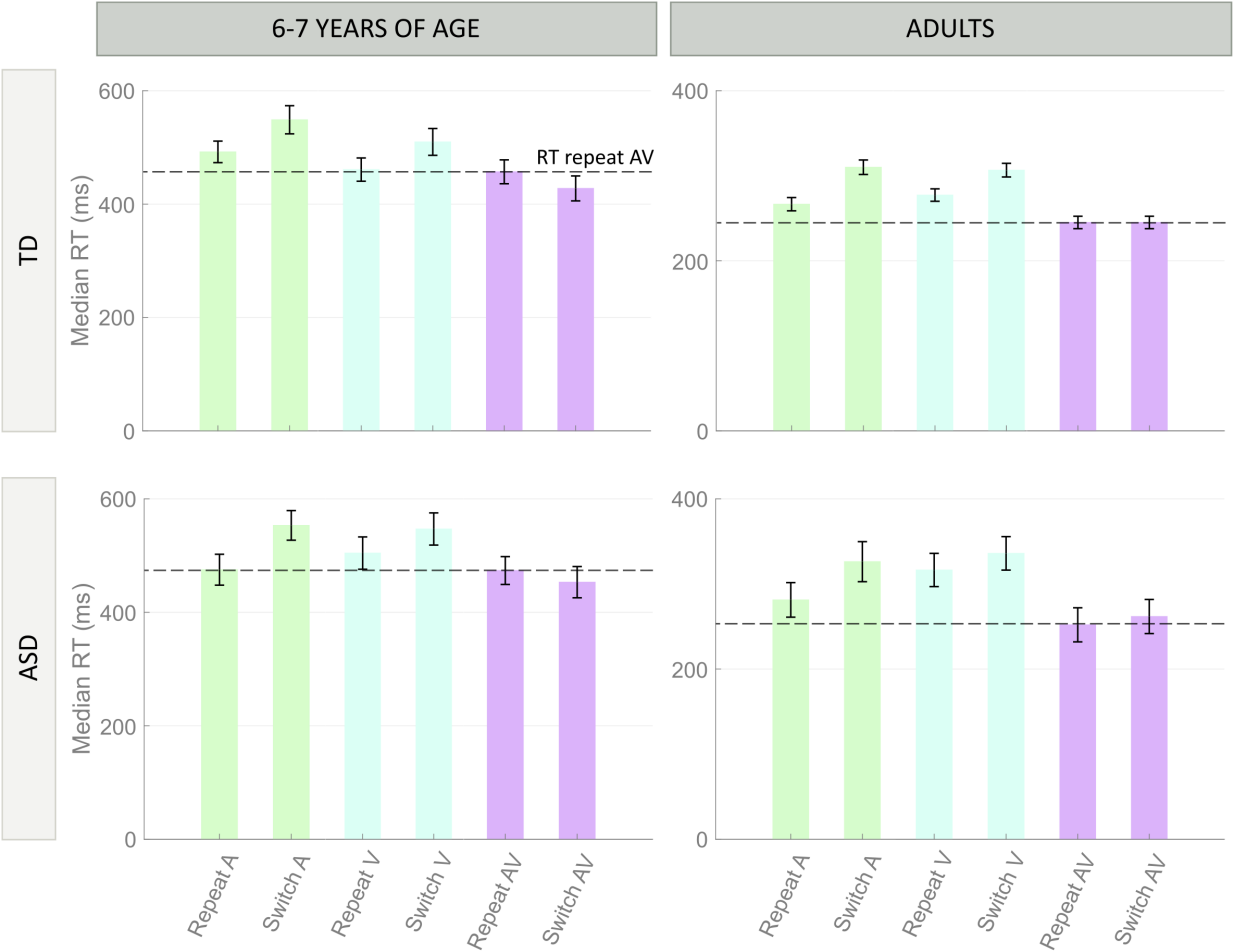
Analysis of unisensory and multisensory RTs for both TD and ASD children of 6–7 years of age. In TD children of 6–7 years of age, RTs in the multisensory repeat trials were comparable with RTs in the visual repeat condition. In ASD children of 6–7 years of age, RTs in the multisensory repeat trials were comparable with RTs in the auditory repeat condition. It is evident that multisensory RTs were not faster than the faster of the unisensory conditions, but exactly comparable to it. Moreover, the AV switch condition is the most favorable condition, producing the fastest responses. Vice versa, in TD and ASD adults, AV repeat and switch present comparable RTs, which are faster than any other unisensory condition.

As can be seen in Figure 2, comparing unisensory and multisensory conditions, RT data do not show a multisensory behavioral facilitation for the 6‐ to 7‐years‐of‐age group. Indeed, responses (RTs) were not faster in the multisensory condition compared to the unisensory case. Interestingly, in TD children (Figure 2), RTs in the multisensory repeat trials were comparable to those in the visual repeat condition, which was the fastest unisensory repeat condition. ANOVA with factor of condition (switch vs. repeat) revealed no significant differences between the RTs in the case of repeat AV and repeat V (F1/52 = 1.34, *p* = 0.25). Similarly, in ASD children (Figure 2), RTs in the multisensory repeat trials were comparable with RTs in the fastest unisensory repeat condition, which was the auditory one for this group (F1/34 = 1.79, *p* = 0.19). Moreover, for both groups, multisensory switch RTs are numerically faster than the RTs in the multisensory repeat condition, even if not statistically relevant (F1/52 = 8.82 × 10^−4^, *p* = 0.98 for TD and F1/34 = 0.16, *p* = 0.69 for ASD). This is not true for the unisensory conditions, where, for both groups, unisensory repeat is always faster than the unisensory switch configuration. Although these differences did not reach statistical significance, the trend was consistent across modalities and groups. In the TD group, RTs were faster in repeat compared to switch trials both for auditory stimuli (F1/52 = 1.83, *p* = 0.18) and visual stimuli (F1/52 = 2.43, *p* = 0.13); similarly, in the ASD group, repeat trials elicited numerically faster responses than switch trials for both modalities (auditory: F1/34 = 0.81, *p* = 0.38; visual: F1/34 = 0.39, *p* = 0.54).

These results, paired with the synaptic architecture and the neural mechanisms underlying the Modality Switch Effect, identified previously (Cuppini et al. 2020), suggest that in the AV repeat condition, participants did not present any multisensory benefit; the preceding stimulus elicits a greater inhibition than the AV switch configuration.

Contrasting children's RT data with adult RTs obtained from the same experimental paradigm (Figure 2), a similar pattern emerges in unisensory trials across both groups, while the behavior in multisensory conditions differs markedly. Indeed, although absolute RTs are considerably slower in children compared to adults, both groups exhibit a modality switch effect in unisensory trials: repeat trials elicit faster responses than switch trials in children (F1/178 = 2.54 × 10^−2^, *p* = 5.08) and in adults (F1/386 = 1 × 10^−4^, *p* = 15). However, only in the adult age‐group does the AV repeat condition yield the overall fastest RTs (faster than all the unisensory RTs, F1/289 = 7 × 10^−4^, *p* = 11.81), with AV switch trials producing RTs comparable to the AV repeat configuration (F1/192 = 0.66, p = 0.19). This would suggest that while unisensory processing does not differ between children and adults, for both switch and repeat, the ability to process multisensory input matures and changes substantially with age, suggesting that learning is occurring to integrate and exploit multisensory information.

### 3.2 Comparison Between Different Neural Architectures and Mechanisms

These observations led us to implement and test two alternative model configurations (Figure 1) to explore the best fit for the children's data. In the “Independent default configuration” (Figure 1B), the two unisensory pathways do not interact reciprocally, as described in Section 2. In this way, the unisensory components of a cross‐sensory event are processed separately, as the model is characterized by independent unisensory processing, and multisensory integration occurs at the level of the output region M. The “Competitive default configuration” (Figure 1A), instead, differs from the previous one for the presence of direct inhibitory connections between the unisensory input regions, implementing an early cross‐sensory competition. In this case, sensory modalities interact as soon as the early stages of sensory processing, in a competitive manner, and then converge to the output layer, where they are integrated.

To quantitatively compare the two network architectures in reproducing empirical data, we computed the MRE index for each experimental condition, both for the empirical RTs and for the RTs simulated by the two model configurations (Table 1). The results show that the Competitive configuration consistently provides MRE values closer to the empirical data than the Independent configuration. In the TD group, the empirical MRE was very low in the repeat condition ($8.5\cdot 10^{-1}$), and the Competitive model reproduced this pattern more accurately (2.9) than the Independent model, which overestimated the enhancement substantially (11). In the switch condition, both models produced values close to the empirical MRE (16), although the Competitive configuration remained slightly more accurate (15 vs. 21). A similar trend was observed in the ASD group. In the repeat condition, the empirical MRE was again very small ($3.1\cdot 10^{-1}$), closely matched by the Competitive model (4.7), while the Independent model largely overestimated the effect (13). In the switch condition, both models approximated the empirical MRE (17), but the Competitive model again provided a more accurate estimate (17 vs. 25).

**TABLE 1:** MRE values for experimental and simulated data across conditions.

	Empirical data	Competitive model configuration	Independent model configuration
${\mathrm{MRE}}_{\mathrm{TD},\;\text{repeat}}$	$8.5\cdot 10^{-1}$	2.9	11
${\mathrm{MRE}}_{\mathrm{TD},\;\text{switch}}$	16	15	21
${\mathrm{MRE}}_{\mathrm{ASD},\;\text{repeat}}$	$3.1\cdot 10^{-1}$	4.7	13
${\mathrm{MRE}}_{\mathrm{ASD},\;\text{switch}}$	17	17	25

*Note:* MRE values are reported separately for repeat and switch trials and for TD and ASD groups. For simulated data, values obtained from both the Independent and Competitive network architectures are shown.

**FIGURE 3:**
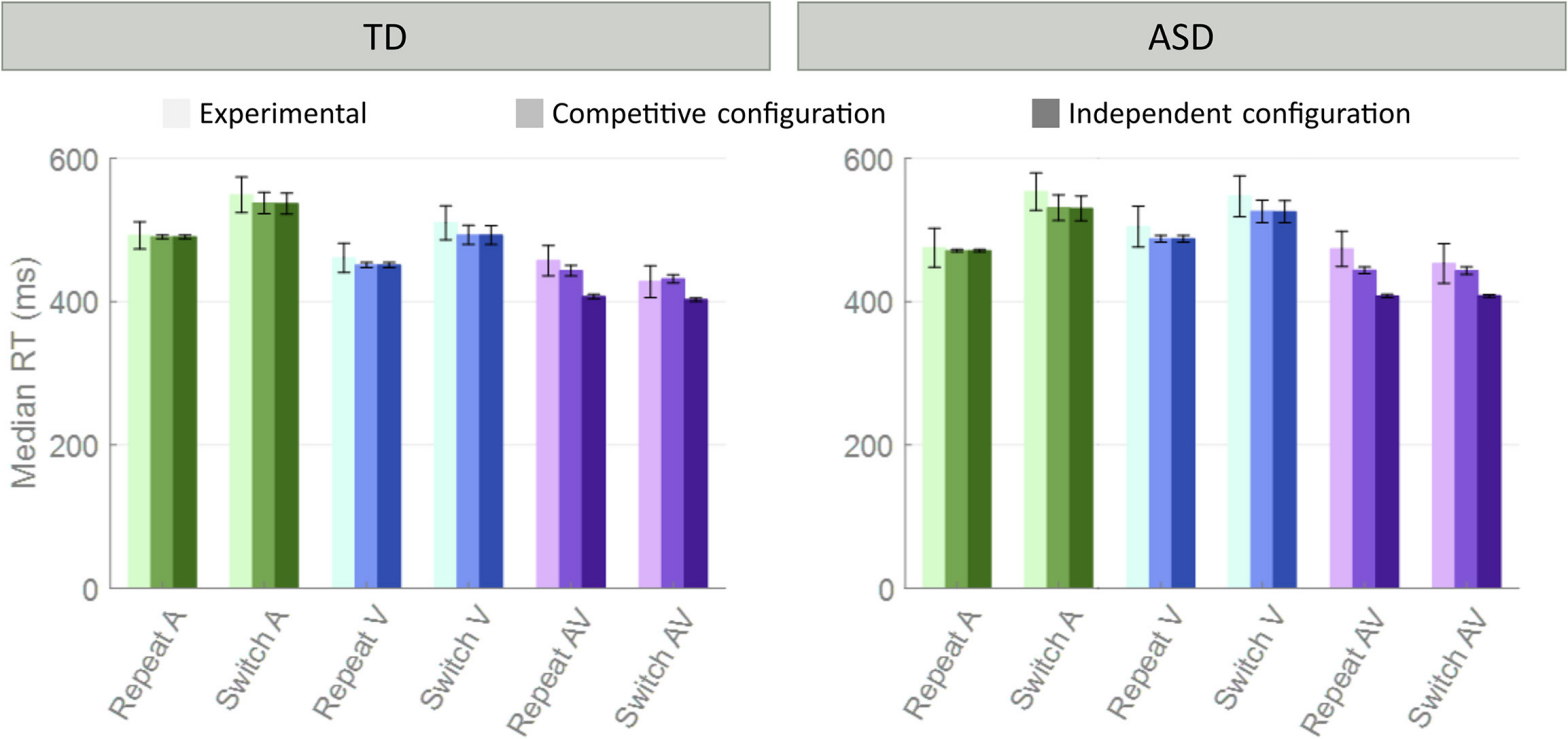
Comparison between the two architectures and empirical data. Cross‐modal competition: the model implementing direct inhibitory synapses between the unisensory input regions is able to reproduce the empirical RTs from Crosse et al. (2022) in every stimulus configuration, for TD and ASD populations. In this configuration, the RTs in multisensory repeat and switch trials were comparable with RTs in the visual repeat condition. No Cross‐modal Interaction: the model without direct inhibitory competition is able to reproduce the empirical RTs from Crosse et al. (2022) only in case of unisensory conditions, but in case of multisensory stimuli it produces faster RTs than the experimental results.

**FIGURE 4:**
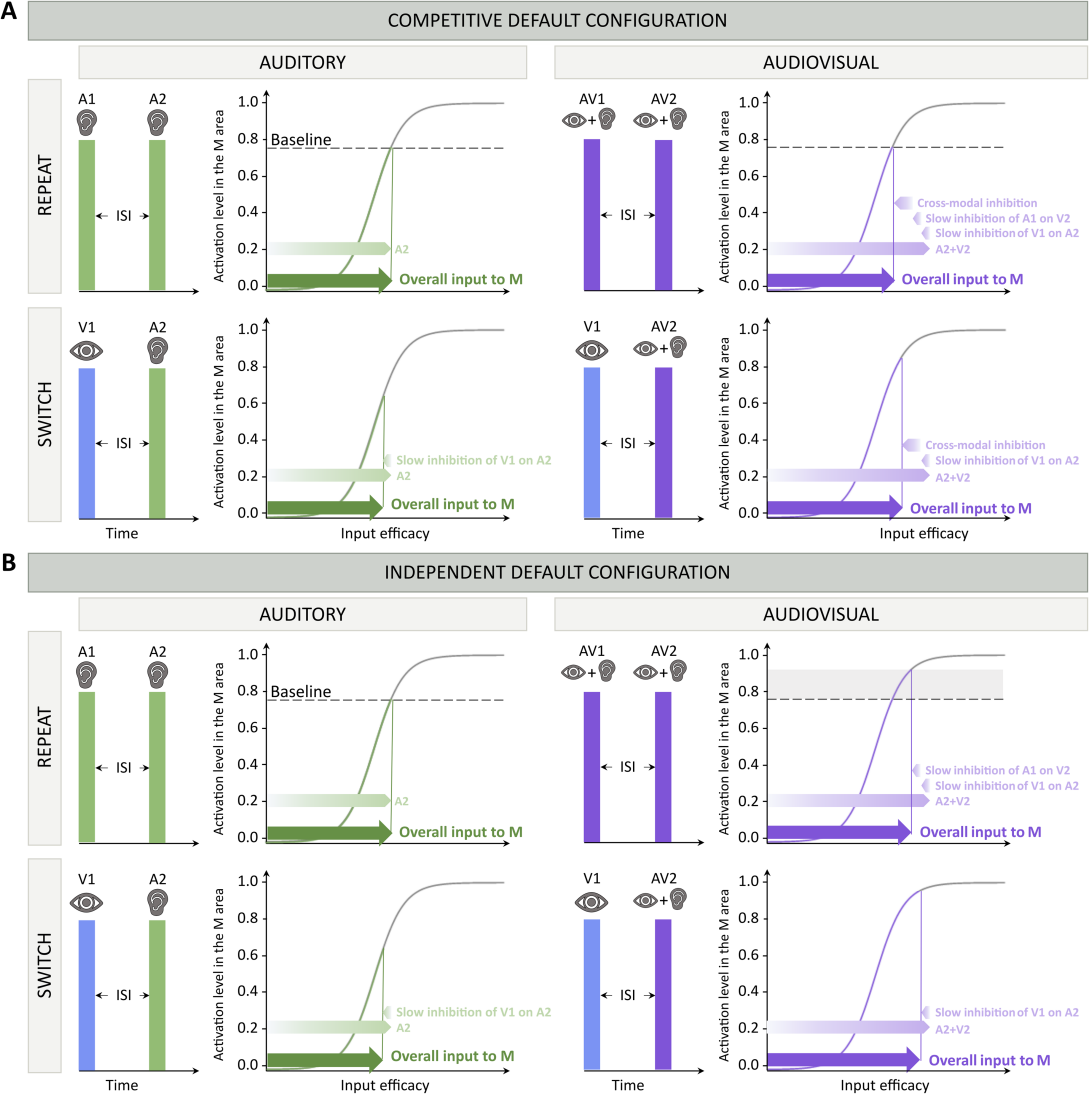
Contribution of input components on the stimulation of unisensory auditory and visual regions. (A) Cross‐modal competition. In the case of unisensory repeat (A1 → A2 in this case) the activity elicited in the output region (colored part of the sigmoidal function) is only determined by the contribution of the unisensory input (green arrow). In the case of multisensory repeat (A1V1 → A2V2), three inhibitory contributions are subtracted from the activity elicited in the M region by the A2V2 stimulus (violet arrow). Indeed, in this condition, two long‐term inhibitory contributions (light violet arrows), that is, that of A1 on the processing of V2 and that of V1 on the processing of A2, come to play. Moreover, also the rapid crossmodal competition between A2 and V2 (dark shaded violet arrow) is present. In the unisensory switch condition (V1 → A2 in this case), the first stimulus (V1) brings in an inhibition (light green arrow), which acts on the processing of the incoming stimulus (A2), thus reducing the activity elicited in the M region. In the multisensory switch condition (V1 → A2V2 in this case) the situation is similar to that described in the multisensory repeat condition, but only one long‐term inhibitory contribution (that of V1 on A2) is present. (B) No cross‐modal interactions. In this case, because of the absence of cross‐modal competition, the activity elicited in the output region is higher in the multisensory repeat condition than in the unisensory repeat condition. Multisensory repeat RTs would be faster compared to the unisensory repeat RTs (the shaded grey area represents the difference between the activation level achieved in the two conditions), which is in contrast with the experimental RTs data. Similarly, also the RTs in the multisensory switch condition would be facilitated compared to the unisensory case, which is not in agreement with the empirical RTs.

As shown in Figure 3, both architectures did a good job of reproducing the empirical results in the unisensory stimulations: The simulated RTs are comparable to the data from Crosse et al. (2022) both for repeat and switch unisensory conditions. This is true for the simulated TD and ASD populations. Nevertheless, only the “Competitive default configuration” is able to reproduce the multisensory AV conditions. On the other hand, the “Independent default configuration” failed to simulate the empirical TD and ASD data because it predicts faster RTs both in the case of AV repeat and AV switch input configurations. As shown in Figure 4B, this latter result is the direct consequence of integration at the level of the output region M. When different senses are stimulated, this region receives converging feedforward synapses from the unisensory regions, carrying the results of the independent processing of the unisensory components, thus bringing together the inputs. This drives a higher response than the unisensory condition, thanks to the non‐linear function simulating the integrative mechanism. Thus, even if, in the input layer, the unisensory processing is carried out independently for different sensory modalities, its result would be combined and integrated at the level of the output region, producing a beneficial effect compared to the unisensory case.

In turn, the reason why only the “Competitive default configuration” (Figure 4A) was able to simulate and explain the behavioral data is the early cross‐sensory competition, implemented through the direct inhibitory synapses between the unisensory input regions. In this configuration, the cross‐sensory competitive mechanism (mediated by the inhibitory cross‐sensory synapses and affecting early sensory processing) balances the integration occurring in the M region (mediated by the excitatory feedforward connections projecting onto it), nullifying any multisensory facilitation, as shown in Figure 4A. In this figure, results in unisensory and multisensory repeat and switch conditions are compared and explained in terms of the inputs contributions to the elicited activity in the output area. In the case of unisensory (A) repeat, the activity is produced only by a single excitatory contribution, A, coming from the auditory sensory processing in the corresponding input region. In the case of AV repeat, instead, the overall contribution to the output is given by (1) the excitatory effect of the multisensory input components, A and V, (2) the inhibition produced by the direct cross‐sensory synapses between the input regions (“cross‐sensory inhibition” in Figure 4), and (3) the contribution of inhibitory feedback from the unisensory interneurons, generated by the preceding AV input (“Long‐term inhibitions” in the figure). The two inhibitory contributions balance out the integration of the unisensory components in area M, so that the response of the network in the case of AV repeat is equivalent to the unisensory repeat condition. In the case of AV switch, since the preceding input is unisensory, in the network there is only one “Long‐term inhibition,” and not two as in the AV repeat. This reduces the inhibitory components, generating an overall higher excitation targeting the output region and eliciting a corresponding faster RT. All of these balancing effects are missing in the “Independent default configuration.”

### 3.3 Model‐Based Characterization of TD and ASD Differences

Stronger cross‐sensory inhibition emerged as a necessary feature of the network architecture when tuning model parameters to fit the ASD group, suggesting increased cross‐modal competition as a distinguishing characteristic of the default sensory configuration in ASD. Although no statistically significant differences were observed between the audiovisual RTs of TD and ASD children, it was not possible to reproduce the behavioral profiles of both groups using a single, shared set of model parameters. Instead, adjusting only one key parameter—the strength of cross‐modal inhibitory projections—allowed us to capture both unisensory and multisensory RTs across the two populations.

### 3.4 ISI Effect and Inter‐Subject Variability

**FIGURE 5:**
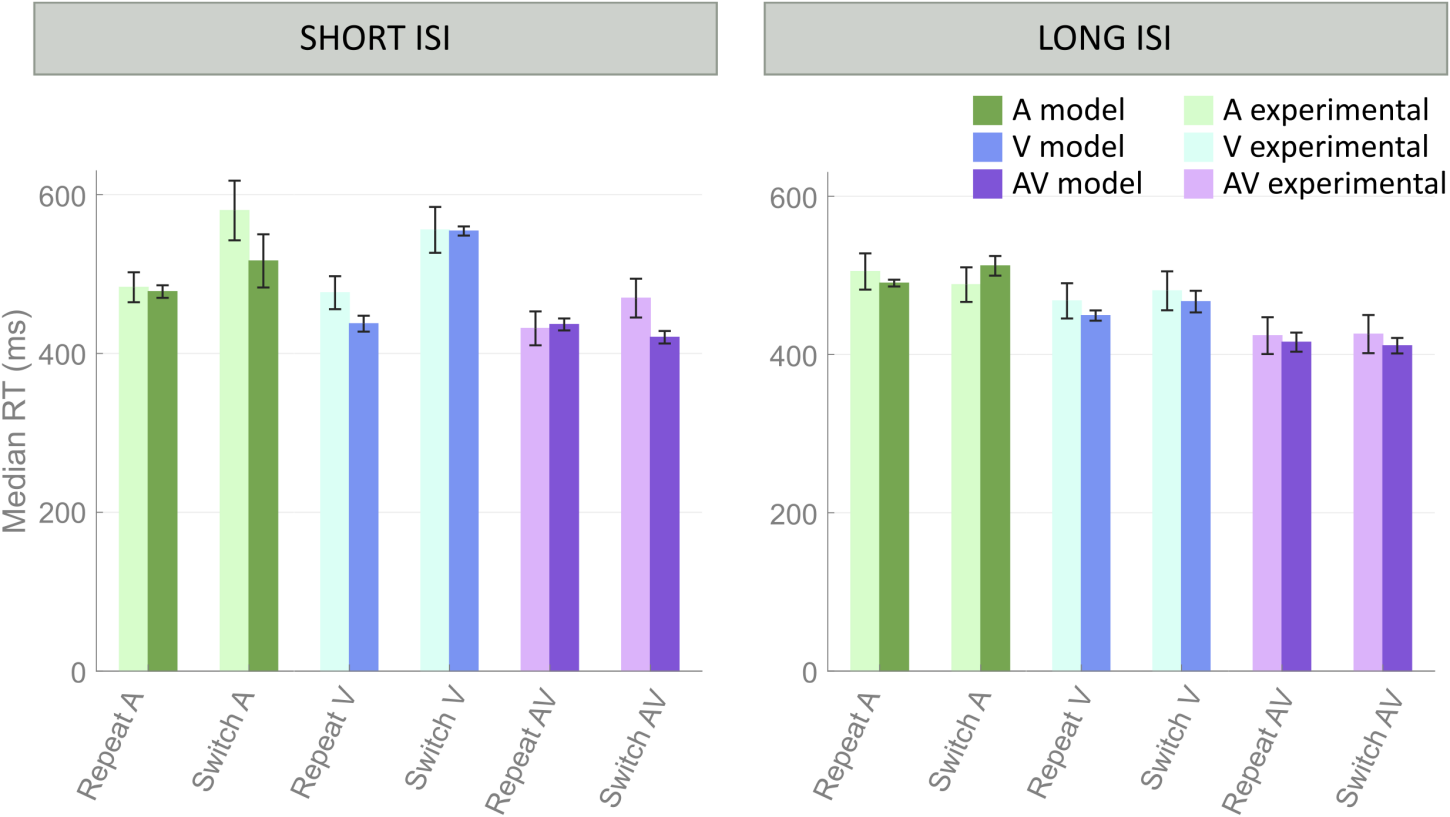
Effect of ISI manipulation. Simulated RTs from the Competitive default configuration are shown for TD children, under Short ISI (1–1.5 s) and Long ISI (2.5–3 s) conditions. Simulated RTs are compared with the empirical ones collected from Crosse's and colleagues under the same experimental conditions. Error bars indicate the standard error of the mean. The model closely replicates the empirical RTs, supporting the validity of the Competitive configuration in capturing the behavioral effects of ISI manipulation.

Figure 5 shows the results obtained for the TD group when manipulating the temporal discrepancies between the stimuli. We analyzed the model's behavior when stimuli were (1) closer in time than the mean ISI, with an ISI randomly selected from a uniform distribution between 1 and 1.5 s (Short ISI), and (2) distant in time more than the mean ISI, with ISIs chosen randomly between 2.5 and 3 s (Long ISI). As shown in Figure 5, the model satisfactorily reproduces the two conditions. These results confirm those previously identified in adult populations (Cuppini et al. 2020): The preceding input exerts an inhibitory effect on the following one. This inhibition lowers for increasing temporal discrepancies (longer ISIs), since it is mediated by inhibitory feedback projections characterized by a slow dynamic, as discussed in our previous paper.

For clarity and brevity, only results from the Competitive configuration for TD children are presented in the main text. Results for the ASD group—showing comparable trends—are provided in Figure S1. Additionally, simulation outcomes from the Independent configuration for both TD and ASD populations are presented in Figure S2. These results further confirm that the Competitive model outperforms the Independent model, more accurately capturing the effects of ISI modulation both qualitatively and quantitatively.

Finally, as shown and discussed by Crosse et al. (2022), even if they found a consistent behavior among participants belonging to the same age group, in terms of RTs and Switch Costs, they also highlighted the great variability of the detected RTs for each stimulus configuration among these participants. To further test and validate the model and the identified basal structure, we sought to reproduce this inter‐individual variability. In a past study (Cuppini et al. 2020), we identified the feedforward synapses and the inhibitory feedback projections as the two mechanisms responsible for different participants' RTs. In line with that analysis, we modified the effectiveness of these synapses by adding a random component to their basal values. This component was generated from a uniform distribution, as detailed in the methods section. Each value of these parameters can be considered representative of a different simulated participant. The mean RTs for each stimulus configuration were calculated for each simulated participant over 100 repetitions per condition. To simulate a physiological input variability among these repetitions, the strengths of the auditory and visual input stimuli in each individual participant were chosen from a uniform distribution (as described previously).

**FIGURE 6:**
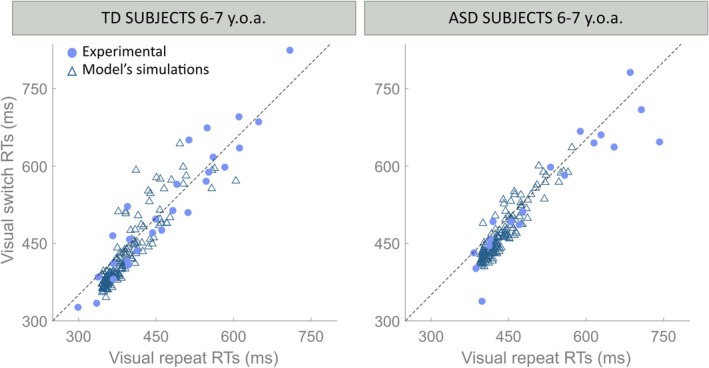
Intersubject variability. Simulated mean RTs are compared with each subject's mean RTs, in the case of Visual Repeat versus Switch configurations. In each panel, the feedforward synapses Wm and synapses L of the inhibitory feedback were randomly varied (uniformly sampled) within the same range [0–10]. The x‐axis reports the RTs in the case of the Repeat condition, the y‐axis the RTs to the Switch condition. Empty triangles represent the mean RTs of the “simulated” subjects, blue dots the subjects' mean RTs, from Crosse et al. (2022). The diagonal black line in each panel represents conditions in which switch RTs are equal to repeat RTs (no switch cost); thus, the vertical distance between data points and the diagonal represents the mean switch cost for each “simulated” or “real” subject.

In Figure 6, as an example of the effect of varying the effectiveness of these connections, we compared the experimental data from Crosse and colleagues to the model results in the case of visual repeat and switch conditions. As shown, introducing those noisy components on the feedforward and feedback synapses allowed us to reproduce the distribution of the RTs for the TD and ASD populations, further validating the identified competitive default architecture of the model. Similar results (not shown here for briefness) have been found for auditory and multisensory conditions.

## 4 Discussion

In the seminal works of Piaget (Piaget 1952), the author postulated that the naïve state of the brain was characterized by unrelated processing of different sensory cues, and only direct interaction with objects and their sensory features and contents would allow the development of sensory integration. On the diametrically opposed end of the theoretical spectrum, Gibson forwarded the differentiation process of sensory perception, where, starting from a default state characterized by an undifferentiated sensory system at birth (Gibson 1966), sensory‐specific experiences emerge due to interaction with the environment. The empirical data are more aligned with Piaget's proposal, supporting the early emergence of multisensory processing that is refined across development. Animal studies show that multisensory neurons, that is, neurons that are sensitive to input from more than one sensory modality, are present at birth (Wallace and Stein 1997, 2001), although these appear to require experience with the environment to exhibit integrative (non‐additive) responses (Wallace 2004; Xu et al. 2014; Yu et al. 2010). In humans, recognition of a causal relationship between multisensory inputs has been reported in infancy (Lewkowicz and Ghazanfar 2009; Muentener et al. 2012), but there is also ample evidence that multisensory processing and the ability to optimally integrate multisensory cues are refined as a function of environmental interactions (Bruns and Röder 2023; Lewkowicz and Ghazanfar 2009), and that this process is highly protracted (Burr and Gori 2012; Gori 2015; Gori et al. 2011; Gori, Giuliana, et al. 2012; Gori, Sandini, et al. 2012). Thus, multisensory integration undergoes an extended maturational process that is heavily influenced by experience, and that appears to extend, depending on the task, through adolescence. Nevertheless, the neural architecture and the mechanisms characterizing the first few years of life, regarding multisensory abilities, and how this maturation process takes place are still unknown.

Recent empirical findings from animal models (Yu et al. 2019) suggest that individual senses interact with each other already in the naïve brain, but in a competitive manner. It is as if the different components of a multisensory stimulus are produced by independent events and compete for the same cognitive resources. Then, only an appropriate multisensory experience would shift this default competitive state toward facilitation, with the effectiveness of crossmodal excitatory synapses overcoming the inhibitory effect of the default competition, thereby developing integrative abilities.

Here we used a computational modeling approach to test between two neurofunctional explanations for developmental changes in unisensory and multisensory processing. We analyzed behavioral data recently reported by Crosse et al. (2022) from a simple audiovisual RT task. This paradigm, implemented to study the development of multisensory integration, provides valuable data for testing the underlying basic neural computation driving the observed behaviors.

Focusing first on the data from the youngest age group of 6–7 years of age, we observed that the RTs, overall, are slower compared to the adult population. Moreover, in multisensory conditions, specifically in a Repeat configuration, RTs are not significantly faster than those in the unisensory Repeat conditions (see Section 3.1). This observation suggests that at this young age, the brain may not effectively utilize the redundant information carried by the different senses, indicating that integrative abilities are still developing. From these observations, we can draw two key inferences. First, the neural mechanisms engaged by a simple reaction‐time task are not yet fully developed, or are different and less effective, in young children (Brandwein et al. 2011; Moore and Linthicum 2007; Ponton et al. 2000). Second, the ability to process multisensory stimuli is not yet fully mature.

The absence of a faster response to a bisensory configuration compared to the fastest of its unisensory components indicates different possible sensory processing scenarios. One possibility is characterized by independent unisensory pathways in the brain that do not interact at any level. In this case, only the fastest or most reliable pathway would trigger a cognitive or motor response from higher‐order regions. Another possibility is that sensory processing could involve a competitive relationship between the two sensory modalities, where each inhibits one another, resulting in a response that is not faster than what independent sensory modalities would produce on their own. To understand which of the previous hypotheses is more plausible, we developed two alternative neural networks, based on different neural mechanisms. The first network replicates the mechanism and synaptic architecture associated with the “independent parallel processing” hypothesis, where different sensory modalities operate in parallel and independently of each other. We refer to this as the “Independent Default Configuration.” The second network aims to represent the “competitive” hypothesis, where sensory modalities interact from birth but in a competitive manner. This is referred to here as the “Competitive Default Configuration.”

It is worth noting that the neural mechanisms implemented in both models are identical to those employed by Cuppini et al. (2020) to simulate the temporal profile of sensory processing in the adult human brain, for both unisensory and multisensory stimuli. The only difference lies in the direct cross‐sensory connections between the input regions. In the adult condition, excitatory connections, which have been strengthened through experience, dominate, as shown in the previously described adult implementation (Cuppini et al. 2020); earlier in development, connections between the sensory cortices can be either dominated by inhibitory connections, like in the case of “Competitive Default Configuration,” or ineffective or absent connections, as in the “Independent Default Configuration.”

Using both models, we simulated the experimental paradigm employed by Crosse et al. (2022) and compared the results of our simulations with the empirical data. The simulations aided us in identifying the structure that could most effectively reproduce the dataset, thus providing insights into the most plausible architecture describing the default configuration and its underlying mechanisms.

Our analysis revealed the “Competitive Default Configuration” as the architecture best capable of reproducing the empirical data. This architecture implies that, in the default configuration, the brain not only fails to utilize redundant sensory information in the case of multisensory stimuli, but also treats stimuli from different sensory modalities in a competitive manner. This suggests that the default configuration is inherently competitive in nature. The RT data collected by Crosse and colleagues support this idea. Their data not only indicate the absence of a multisensory facilitation in young children, as multisensory RTs were not faster than the fastest unisensory condition, but they also suggest the existence of competition between sensory modalities in these children. The fact that behavioral responses in the multisensory repeat condition are comparable with the fastest unisensory repeat condition suggests that, in the case of audiovisual stimulation, the two sensory channels compete in a way that only one of them can drive the behavioral response and generate the observed RTs. Furthermore, since in the adult population, multisensory conditions elicit robustly faster RTs than unisensory stimuli, the default cross‐modal competition, operating between sensory modalities in young children, is likely to be superseded during development by the classical multisensory facilitation, as highlighted by Crosse et al. (2022) and previous analyses involving animal models (Yu et al. 2019) (also see Cuppini et al. 2020 for a computational framework).

Being a biologically plausible computational framework, our model also allows us to speculate on the possible biological substrates of its components. This multisensory output area may correspond to associative cortices, known to support sensory integration, such as the Posterior Parietal Cortex. Indeed, evidence from human intracranial recordings supports the role of the superior parietal lobule in audiovisual multisensory integration (Molholm et al. 2006).

Regarding the competitive layer, we speculate its implementation through higher order regions such as the medial Prefrontal Cortex, Posterior Cingulate Cortex, or secondary higher order sensory cortices. Huang et al. (2015) suggested that these regions may be involved in the competition between A and V sensory modalities in a simple RT experiment; Hairston et al. (2008) reached a similar conclusion in the case of an auditory temporal order judgment task. However, it is also plausible that such inhibitory mechanisms are instantiated within early sensory cortices via local interneuron circuits. Given that long‐range cortical projections are typically excitatory, the observed cross‐modal inhibition could arise via a disynaptic pathway: for example, pyramidal neurons in one sensory modality (e.g., auditory cortex) could activate, through long‐range excitatory connections, local inhibitory interneurons in a different sensory modality (e.g., visual cortex), which in turn inhibit pyramidal neurons within their own region. This possibility suggests that both excitatory and inhibitory elements would be located within early sensory areas. Therefore, the control layer implemented in our model may not require a separate higher‐order substrate but could instead reflect distributed interactions embedded in early sensory processing networks. This scheme is consistent with the evidence that, in the cortex, the building block of sensory information processing is constituted by microcircuits of interconnected excitatory pyramidal cells and inhibitory interneurons (see Wood et al. 2017 for a review).

A growing body of anatomical and functional evidence also supports the presence of direct influence between primary sensory areas, providing a plausible substrate for cross‐modal interactions. Studies in animal models have revealed anatomical projections between core visual and auditory regions and associative areas (Cappe and Barone 2005; Clavagnier et al. 2004; Falchier et al. 2002), while other work has demonstrated modulatory connections among sensory regions (Bizley and King 2008, 2009; Meredith and Allman 2015; Yu et al. 2013).

### 4.1 Model Validation: ISI Effect on Modality Switch Effect

By manipulating the temporal discrepancy between sensory inputs, we assessed the implemented model and its core mechanisms. This allowed us to confirm that the Switch Cost, which is already evident at the age of 6–7, results from the long‐lasting competition among sensory modalities. Additionally, this effect decreases with increasing temporal distance between the two stimuli. These findings, coupled with the analysis of RTs collected by Crosse and colleagues, indicated the presence of this effect even in the developing brain, highlighting a prevailing condition where sensory processing appears to be governed by inhibition and competition. In fact, in the default configuration, our network is characterized by cross‐sensory competition with a fast dynamic, exerting its effect on the current stimulus, and a cross‐sensory competition characterized by a slower dynamic, which imposes an inhibitory effect on the incoming input.

Based on the identified neural architecture, we could predict that, as individuals mature, experience with congruent bisensory stimuli shapes this default configuration. This process would establish cooperative and integrative abilities mediated by cross‐sensory excitatory synapses. Initially, these synapses would counterbalance the inhibitory effect of cross‐sensory competition between unisensory regions. Over time, excitation would prevail over inhibition, leading to the cross‐sensory facilitation that characterizes the adult configuration (as suggested in Cuppini et al. 2020; McIntosh et al. 1998; Raij et al. 2010). In this mature configuration, then, the only remaining cross‐sensory competition is the long‐lasting one, which mediates the switch cost (Cuppini et al. 2020; Shaw et al. 2020).

This process could be interpreted as the neural implementation of the perceptual narrowing mechanism described by Lekowicz and colleagues (Lewkowicz and Ghazanfar 2009), where the narrowing effect observed during childhood development is not due to the loss or pruning of previously effective diffuse synapses, but rather, it is the effect of the maturation of new connections, whose development is determined by postnatal cross‐sensory experience (Lewkowicz and Ghazanfar 2009). These cross‐sensory synaptic arrangements among unisensory areas and their predicted development could also explain results and theories of map alignment (Burr and Gori 2012) and the cross‐sensory recalibration among the senses as the results of multisensory coarse organization in the young brain (Bruns and Röder 2023). In this postulated default configuration, the feedforward synapses would implement a coarse multisensory integration, showing up in the associative output region. The direct, inhibitory, cross‐sensory synapses would implement the lack of specificity in the ability to integrate multisensory cues characterized by common spatial features. With experience, only the excitatory synapses among sensory‐specific neural elements sensitive to the same spatial positions would be strengthened, mediating the spatial alignment of sensory maps and the calibration of sensory processing among the different sensory modalities, and generating a more precise integration of spatially aligned stimuli.

Nevertheless, the current model architecture does not include multiple spatially tuned units within each sensory area; therefore, it does not allow us to formally test this hypothesis against alternative mechanisms, such as synaptic pruning. Future extensions of the model will incorporate spatially selective neuronal populations, enabling a direct comparison between different developmental processes that may underlie the emergence of multisensory specificity and cross‐sensory map alignment.

### 4.2 ASD Simulations

After identifying the most likely default configuration for cross‐sensory interactions, we aimed at characterizing the differences observed in the ASD population. The configuration of the model that successfully reproduced ASD behavior further validated the competitive default configuration. To match the RTs of the ASD population, we found that cross‐sensory synapses needed to be much more inhibitory (i.e., stronger cross‐sensory competition).

Notably, developmental trajectories observed in individuals with ASD, particularly in terms of perceptual abilities, are often delayed compared to those of their TD peers (Beker et al. 2018; Crosse et al. 2022; Foxe et al. 2015; Wakim et al. 2023). Therefore, the state of the autistic brain at a specific developmental stage offers insight into the situation in the typically developing brain at an earlier stage of development. From this perspective, the requirement for stronger crossmodal competition to reproduce the RTs of children with ASD further reinforces the concept of competitive default configuration.

### 4.3 Testable Predictions

These observations prompted us to ask whether this default general competition, governed by fast and slow temporal dynamics, could offer insights into the emergence of the temporal window of integration (TWI) and its alterations in pathological conditions. We wondered whether the TWI, and any anomalies seen in it, might arise from specific crossmodal experience. In this scenario, when multisensory cues are atypically paired in terms of their temporal alignment, it could lead to the development of an atypical TWI and altered temporal integration phenomena (Burr and Gori 2012; Hötting and Röder 2009; Powers et al. 2009; Scarpina et al. 2016). Similarly, crossmodal stimuli with atypical spatial pairings could result in altered spatial integration abilities (Wallace and Stein 2007).

Furthermore, our simulations suggest a distinct pattern of brain activation in the case of multisensory stimulation in adults and children. Specifically, in children, the unisensory regions should exhibit reduced activity (or excitation) in response to multisensory stimuli compared to unisensory conditions. This reduction is attributed to the inhibition mediated by cross‐modal connections. In contrast, in adults, unisensory regions should show increased activity (or excitation) in response to multisensory stimuli compared to unisensory conditions due to the excitation mediated by the now‐excitatory reciprocal crossmodal projections. This prediction aligns with EEG data presented in figure 4, from Brandwein et al. (2011).

### 4.4 Future Directions

To validate and expand upon this theoretical framework, we are currently implementing a training algorithm that simulates the acquisition of MSI in TD and ASD children throughout childhood and adolescence. This would allow us to shed light on if and how sensory experience impacts the acquisition of MSI and the trajectory of its maturation. Additionally, this could suggest potential training paradigms to facilitate MSI maturation in children with ASD.

It is important to acknowledge that the behavioral data from (Crosse et al. 2022), while informative, may not be optimal for directly testing the default state of cross‐sensory interactions. The experimental paradigm used in that study primarily targets correlations and dependencies in unisensory processing across trials—specifically contrasting repeat versus switch modality conditions—rather than examining interactions between auditory and visual components *within* a single trial. However, it is precisely this within‐trial interaction between sensory modalities that is most relevant to the mechanisms proposed in our model. In future work, we plan to test the model more directly by acquiring new behavioral data that systematically manipulate the temporal proximity of the auditory and visual components of the same stimulus. This approach will allow us to probe how cross‐sensory integration evolves as a function of audiovisual alignment or separation, and to assess whether the neural mechanisms underlying these effects can be captured within the same mechanistic framework outlined by our current model.

Additionally, future research will seek to broaden this framework, which is now limited to the temporal domain of integrative abilities. Our goal is to extend it to encompass the spatial domain as well. This expansion will delineate a comprehensive framework capable of describing and analyzing the perception of stimuli characterized by complex temporal and spatial properties. In doing so, we aim to work with inputs that more closely resemble the complexity of real‐world stimuli encountered in our daily lives, rather than relying solely on the experimental stimuli commonly used in empirical research.

## Author Contributions

**Melissa Monti:** conceptualization, formal analysis, methodology, software, visualization, writing – original draft. **Sophie Molholm:** supervision, writing – review and editing. **John J. Foxe:** writing – review and editing. **Cristiano Cuppini:** conceptualization, funding acquisition, methodology, project administration, software, writing – original draft, writing – review and editing.

## Conflicts of Interest

The authors declare no conflicts of interest.

## Peer Review

The peer review history for this article is available at https://www.webofscience.com/api/gateway/wos/peer‐review/10.1111/ejn.70233.

## Supporting information


**Table S1:** Parameters value.
**Figure S1:** Effect of ISI manipulation in the Competitive model configuration. Simulated RTs from the Competitive default configuration are shown for both TD and ASD populations, under Short ISI (1–1.5 s) and Long ISI (2.5–3 s) conditions. Simulated RTs are compared with the empirical ones collected from Crosse's and colleagues under the same experimental conditions. Error bars indicate the standard error of the mean. The model closely replicates the empirical patterns in both populations, supporting the validity of the Competitive configuration in capturing the behavioral effects of ISI manipulation.
**Figure S2:** Long versus short ISI in the independent default configuration. Simulated RTs from the Independent default configuration are shown for both TD and ASD populations, under Short ISI (1–1.5 s) and Long ISI (2.5–3 s) conditions. Simulated RTs are compared with the empirical ones collected from Crosse's and colleagues under the same experimental conditions. Error bars indicate the standard error of the mean. Compared to the Competitive model, the Independent configuration provides a poorer fit to the empirical data in terms of ISI modulation.

## Data Availability

The data used in this study are available upon reasonable request to the corresponding author.
