# Supplementary material for: Is Competition the Default Configuration of Cross‐Sensory Interactions?
Source: Eur J Neurosci. 2025 Aug 26;62(4):e70233. doi: 10.1111/ejn.70233 (PMC12381403; doi:10.1111/ejn.70233)
Supplement: Supplementary file 1 — Table S1: Parameters value. Figure S1: Effect of ISI manipulation in the Competitive model configuration. Simulated RTs from the Competitive default configuration are shown for both TD and ASD populations, under Short ISI (1–1.5 s) and Long ISI (2.5–3 s) conditions. Simulated RTs are compared with the empirical ones collected from Crosse's and colleagues under the same experimental conditions. Error bars indicate the standard error of the mean. The model closely replicates the empirical patterns in both populations, supporting the validity of the Competitive configuration in capturing the behavioral effects of ISI manipulation. Figure S2: Long versus short ISI in the independent default configuration. Simulated RTs from the Independent default configuration are shown for both TD and ASD populations, under Short ISI (1–1.5 s) and Long ISI (2.5–3 s) conditions. Simulated RTs are compared with the empirical ones collected from Crosse's and colleagues under the same experimental conditions. Error bars indicate the standard error of the mean. Compared to the Competitive model, the Independent configuration provides a poorer fit to the empirical data in terms of ISI modulation. [file EJN-62-0-s001.pdf]

Supplementary Material

Mathematical implementation of the network

As described above, each region of the model is described by a single neural element. Every element of the network has been described by means of a first order differential equation, which simulates the integrative properties of the cellular membrane, and a steady-state sigmoidal relationship that simulates the presence of a lower threshold and an upper saturation for neural activation. The saturation value is set at 1, i.e., all outputs are normalized to the maximum. The term "activity" is used to denote the output of each area from now on.

In the following, each element will be denoted with a superscript, r, referred to a specific region of the model ($r = a, v, m, Ia,$ or Iv, where a refers to the auditory input area, v to the visual input area, m to the multisensory output region, $Ia,$ and Iv to the inhibitory auditory and visual neurons, respectively). $u(t)$ and $y(t)$ are used to represent the net input and output of a given neural element at time t, respectively. Thus, $y^r(t)$ and $u^r(t)$ represent the output and the net input of the neural element simulating the region r, respectively. A generic neural element of a region r is described by the following differential equation:

$$\tau^n \frac{dy^r(t)}{dt} = -y^r(t) + F(u^r(t)) \tag{1}$$

where τ is the time constant and $F(u(t))$ represents the sigmoidal relationship:

$$F\left(u^r(t)\right) = \frac{1}{1+e^{-s(u^r(t)-\theta)}} \tag{2}$$

s and θ are parameters which establish the slope and the central position of the sigmoidal relationship, respectively. For the sake of simplicity, in this work all the neural elements are described by using the same parameters (s and θ) and the same time constant (τ^n).

The net input that reaches a specific neural element (i.e., the quantity $u^r(t)$ in Eq. (1)) depends on the region it belongs to.

Input areas

Elements in these regions process separately auditory and visual external stimuli ($r = a, v$). Their net input is the result of three components.

The first, the "external" component, is the unisensory input $e^r(t)$, coming from the external world. The second, the "cross-modal" component, is the input, $c^r(t)$, from the area processing the other sensory modality, transmitted to the target neuron through the cross-modal synapses. This component is present only in the Competitive default configuration of the model, characterized by a direct competition between the input regions (Fig. 1A). The last component is the contribution of the feedback inhibitory synapses, $I^r(t)$, which is the effect of the interneurons excited by the other sensory region that interacts with the target element through inhibitory synapses.

The external input is characterized by its effectiveness I_0^r, and its duration T^r. Assuming a stimulus of sensory modality r ($r = a \; or \; v$):

$$e^r(t) = \begin{cases} I_0^r, & 0 \le t \le T^r \\ 0, & t > T^r \end{cases} \tag{3}$$

In the Competitive default configuration, the cross-modal input, $c^r(t)$, is obtained assuming that each element receives a projection from the region processing the other modality defined as:

$$c^a(t) = W_{av} \cdot y^v(t - \Delta t)$$
$$c^v(t) = W_{va} \cdot y^a(t - \Delta t) \tag{4}$$

where W_{av}, W_{va} are the weights of these reciprocal connections, and Δt is a delay which represents the latency with which cross-modal inputs are exchanged between the two regions.

Finally, the inhibitory input, $I^r(t)$, that a unisensory element receives from the interneuron of the other modality is defined as:

$$l^a(t) = L_a \cdot y^{Iv}(t)$$
$$l^v(t) = L_v \cdot y^{Ia}(t) \tag{5}$$

where $y^{Ia}(t)$ and $y^{Iv}(t)$ are the activities of presynaptic auditory and visual interneurons respectively, and L_a, L_v are the strengths of the inhibitory synapses. In the model, we did not incorporate a delay for the cross-sensory inhibition, because the dynamics of the inhibitory effect has been chosen much longer than any other mechanism of the network (see below *Dynamics of each input component*), so the effect of a delay is already included in the time constant chosen for these projections.

Inhibitory interneurons

Elements in these two regions ($r = I_a, I_v$) are excited respectively by the auditory and visual input areas, and they exchange inhibitory projections, implementing a "winner-takes-all" (WTA) mechanism. Their net input is the result of an excitatory stimulus, $I\_ex^r(t)$, coming from the corresponding unisensory input region through excitatory synapses, and an inhibitory component, $I\_in^r(t)$, produced by inhibitory synapses from the other interneuron.

The excitatory components, $I\_ex^r(t)$, targeting the auditory and visual interneurons are defined as:

$$I\_ex^{Ia}(t) = WI_a \cdot y^a(t)$$
$$I\_ex^{Iv}(t) = WI_v \cdot y^v(t) \tag{6}$$

where WI_a and WI_v are the weights of the excitatory connections from a unisensory input region to its corresponding interneuron element, assumed the same for both sensory modalities ($WI_a = WI_v = WI$).

The inhibitory input, $I\_in^r(t)$, that an interneuron receives from the interneuron of the other modality, through inhibitory synapses, is defined as:

$$I\_in^{Ia}(t) = L_{av} \cdot y^{Iv}(t)$$
$$I\_in^{Iv}(t) = L_{va} \cdot y^{Ia}(t) \tag{7}$$

where $y^{Ia}(t)$ and $y^{Iv}(t)$ are the activities of presynaptic auditory and visual interneurons respectively, and L_{av}, L_{va} are the strengths of the reciprocal inhibitory connections. These symmetrical synapses ($L_{av} = L_{va} = LI$) implement the WTA mechanism between the two areas. Also in this case, as in Eq. (5), we do not include a pure delay for the same reason stated above.

Multisensory output area

This region ($r = m$) receives a net input that is the sum of the stimuli, carried by long-range excitatory synapses, from the auditory and visual input areas.

Its net input, $ex^m(t)$, is defined as:

$$ex^m(t) = \sum_r W_{mr} \cdot y^r(t - \Delta t^m);\ \forall\, r = a, v \tag{8}$$

where W_{ma} and W_{mv} are the weights of the excitatory connections from the unisensory input regions to the multisensory area and Δt^m is a delay, which represents the slightest latency with which stimuli from the input regions are able to generate behavioral responses.

Dynamics of each input component

All previous quantities (Eqs. (3) to (8)) affect the input $u^r(t)$ of the corresponding postsynaptic element via a second order differential equation. By denoting with $o_i(t)$ the output of the differential equation for the generic input source $i(t)$ (described by any of Eqs. (3) to (8)) we have

$$\begin{cases} \frac{d}{dt} o_i(t) = \delta_i(t) \\ \frac{d}{dt} \delta_i(t) = \frac{G_i^r}{(\tau_i^r)^2} i(t) - \frac{2 \cdot \delta_i(t)}{\tau_i^r} - \frac{o_i(t)}{(\tau_i^r)^2} \end{cases} \tag{9}$$

where G_i^r represents the gain and τ_i^r defines the time constant of the dynamics, for each region r, and input component i (Eqs. (3) to (8)). Eq. (9) implements a second-order impulse response with two coincident real poles. This is used frequently in neural modeling to mimic synaptic dynamics (Cuppini et al., 2014, 2020; Jansen & Rit, 1995; Wendling et al., 2002). In the model, in order to reduce the number of parameters, we choose the same values for G_i^r and τ_i^r, for every connection (see Table 1), except two cases: (i) the external stimuli, and, (ii) the feedback synapses implementing the cross-sensory inhibitory mechanism.

According to the previous description, the total input (namely $u^r(t)$) received by a neuron in region r, is computed as follows:

i For the input regions, it is the sum of the external component (Eq. (3)), cross-modal term, if present, (Eq. (4)), and inhibitory feedback (Eq. (5)), filtered through the second order equation (Eq. (9)):

$$u^r(t) = o_e(t) + o_c(t) + o_I(t);\ \forall\, r = a, v \tag{10}$$

ii For the inhibitory interneurons, it is the sum of the excitation from the input region (Eq. (6)), and the effect of the WTA mechanism (Eq. (7)), filtered by Eq. (9):

$$u^r(t) = o_{I\_ex}(t) + o_{I\_in}(t);\ \forall\, r = Ia, Iv \tag{11}$$

iii For the output region, it is the effect of the feedforward excitatory synapses (Eq. (8)), filtered by the differential equation previously described (Eq. (9)):

$$u^m(t) = o_{ex}(t) \tag{12}$$

Parameters assignment

The values of model parameters (Table S1) were assigned according to the criteria summarized below, based on findings reported in the literature. Parameters describing the elements of the network were assumed equal for the two sensory modalities, with the only exception of the external inputs, to reduce the number of ad hoc assumptions. Moreover, the values of the synaptic weights were manually adjusted to achieve the best fit between the model and behavioral data reported in Crosse et al. (2022), specifically, to reproduce experimental data of 6-7 year-old subjects. To ensure

biological validity, parameters tuning was guided by constraints from the literature, including known sensory latencies and activation dynamics, as well as prior modeling studies using similar architectures (Cuppini et al., 2012, 2018; Cuppini, Magosso, et al., 2011; Magosso et al., 2012; Ursino, Cuppini, et al., 2017). To ensure that the final parameter set did not simply overfit one specific dataset or configuration, we also validated the model on additional experimental paradigms. In particular, we tested the model's ability to account for behavioral effects under different ISI values and across inter-individual variability, demonstrating that it could robustly capture the modulation of modality switch costs without further tuning.

Parameters of individual neurons

The central abscissa, θ, was assigned to have negligible neuron activity in basal conditions (i.e., when the input was zero). The slope of the sigmoidal relationship, s, was assigned to have a smooth transition from silence to saturation in response to external stimuli. The time constant agreed with values (a few ms) normally used in deterministic mean-field equations (Ben-Yishai et al., 1995; Treves, 1993).

External input $e^r(t)$

Physiological evidence shows that in the brain, auditory processing is faster, and auditory cortical neurons exhibit shorter latencies (e.g., Recanzone et al., 2000) than neurons in the visual cortex (Maunsell & Gibson, 1992). As we did in a previous model (Cuppini et al., 2014), in this network the visual input region receives external stimuli described by a slower time constant, compared with the auditory ones. This is mimicked by setting $\tau_e^a < \tau_e^v$ in Eq. (9). The values of parameters τ_e^a and τ_e^v have been assigned to reproduce the temporal evolution of the process of an auditory and a visual stimulus in the early cortical areas. In particular, τ_e^a is given so that the auditory processing presents the faster dynamics, and the auditory area is activated by an auditory input 25-30 ms after the stimulus. Since two time constants represent the time needed for the activity in the input regions to reach 90% of its steady-state level, in response to a step input, we assume τ_e^a = 15 ms. For what concerns the visual area, τ_e^v is assigned so that a visual stimulus produces a detectable response in the visual area 45-50 ms after its onset; hence τ_e^v = 25 ms has been chosen. It is worth noting that these are the only differences between the two sensory processing pathways (auditory and visual); all other parameters are assumed equal for the auditory and visual branches of the network.

The strength of the external visual and auditory stimuli (parameters I_0^v and I_0^a) are chosen so that the overall input elicits a response, in the input regions, in the linear part of the sigmoidal static characteristic (i.e., a little below saturation).

Synaptic connections

The effectiveness of the feedforward connections targeting the M region (W_{ma} and W_{mv}) is set so that an effective unisensory stimulus of sufficient strength (i.e., able to lead input areas close to saturation) evokes an activity in lower part of the linear region of the sigmoidal activation function (i.e., about 30% of the maximum activity in this area). These parameters were set to reproduce data acquired by Crosse et al. (2022) in children of 6-7 years of age, for TD and ASD simulations.

Feedforward connections to the M regions are characterized by a fast dynamic (τ_i^r = 50 ms; with $i = m$) and by a delay $\Delta t^{\,m}$, to discriminate fast outliers. In particular, previous studies considered RTs faster 100 – 150 ms as anticipatory responses (Crosse et al., 2022; Molholm et al., 2020). Therefore, we assigned to $\Delta t^{\,m}$ an average value between those found in the literature ($\Delta t^{\,m}$ =120 ms).

Excitatory feedforward connections to interneurons, WI, are characterized by a value chosen so that even a small activity in the input layer is able to elicit an activity in the interneuron of the

corresponding modality, activating the cross-sensory inhibitory mechanism. This element is characterized by a fast dynamic (τ_i^r = 15 ms; with $i = I_{ex}$).

Interneurons reciprocal inhibition, $I\_in^r(t)$, implements the WTA competition. The effectiveness of the reciprocal synapses, LI, is chosen high enough so that the "winner" interneuron is able to turn off almost completely the "loser" element. This element is characterized by a fast dynamic as well (τ_i^r = 15 ms; with $i = I_{in}$).

Behavioral data (Crosse et al., 2022) show that strongest cross-sensory inhibitory effect occurs for ISIs as short as 1000 ms, but this effect decays slowly for longer time intervals between the stimuli. To simulate this result, the feedback inhibitory synapses in the model have slow dynamics, implemented by time constants for the feedback projections as great as to 180 ms ($\tau_l^a = \tau_l^v$). In fact, with such a time constant, the inhibitory component provides a significant contribution to the input regions of the other modality after almost 1000 ms after stimulus presentation. In summary, the input regions are activated by an external stimulus after almost 50 ms (time constants 15-25 ms); through the excitatory projections, the interneurons show a non-null activity after 100-120 ms, and a peak activation between 250 ms and 300 ms; then the chosen inhibitory dynamics add 540 ms (approximately three time constants) before the feedback inhibitory component reaches its maximal effect on the unisensory input regions.

As stated above, the values of the inhibitory feedback connections (L_a, L_v), as well as parameters of cross-modal synapses, in the Competitive default configuration, were set to reproduce data acquired by (Crosse et al., 2022) in children of 6-7 years of age, for TD and ASD simulations.

The parameters of cross-modal synapses are selected to reproduce empirical findings by Raij et al. (2010). These authors, combining MEG and fMRI recordings, studied cross-modal activations and audio-visual interactions in the primary auditory cortex (A1) and in the primary visual cortex (V1) at very early post stimulus latencies. To simulate (Raij et al., 2010) results we used the input to the neurons (i.e., quantity $u(t)$ in Eq. (1)) since this is indicative of field potentials, detected through EEG or MEG techniques, and/or synaptic metabolic activity, detected through fMRI. The dynamics of the reciprocal cross-modal projections have been chosen fast and symmetrical for both sensory modalities. Their time constant (τ = 15 ms) and the delay in cross-modal synapses, Δt = 16 ms, simulating the latency with which the influence of a unisensory stimulus was detected in the area processing the other sensory modality, are selected so that the cross-modal component produces an effect with a rapid time course, affecting "the other region" after further 30-40 ms.

For all the above elements, G_i^r the values are chosen so that the elicited activity in the post-synaptic elements is in the linear portion of the sigmoidal relationship: as shown in Table 1, G_i^r = 75 for every synapse ($i = e, c, I\_ex, I\_in, m$), except for the inhibitory feedback ($i = l$) where G_l^r = 750.

***Table S1**- Parameters value.*

**Neurons**			
$\theta = 25$		$s = 0.3$	$\tau = 3\ ms$
**Inputs**			
$G_i^r = 75;$	$\tau_k^r = 15\ ms;$	$\tau_e^a = 15\ ms$	$\tau_e^v = 25\ ms$
$i = e, c, I_e x, I_i n, m$	$k = c, I_e x, I_i n, m$	$T^a = 60\ ms$	$T^v = 60\ ms$
		$I_0^a = 2.65\ (\pm noise\%)$	$I_0^v = 2.65\ (\pm noise\%)$
**Inhibition**			
$G_l^r = 750;\ r = a, v$		$\tau_l^r = 180\ ms;\ r = a, v$	

**Synapses**			
$L = 3$	$L_{aIv\_TD} = 0.09$	$W_{av\_TD} = -0.6$	$W_{ma\_TD} = 1$
$WI = 1.4$	$L_{vIa\_TD} = 0.07$	$W_{va\_TD} = -0.6$	$W_{mv\_TD} = 1.72$
	$L_{aIv\_ASD} = 0.13$	$W_{av\_ASD} = -0.8$	$W_{ma\_ASD} = 1.1$
	$L_{vIa\_ASD} = 0.05$	$W_{av\_ASD} = -0.8$	$W_{mv\_ASD} = 1.3$
		$\Delta t = 16\, ms$	$\Delta t^{m} = 180\, ms$

Supplementary Figures

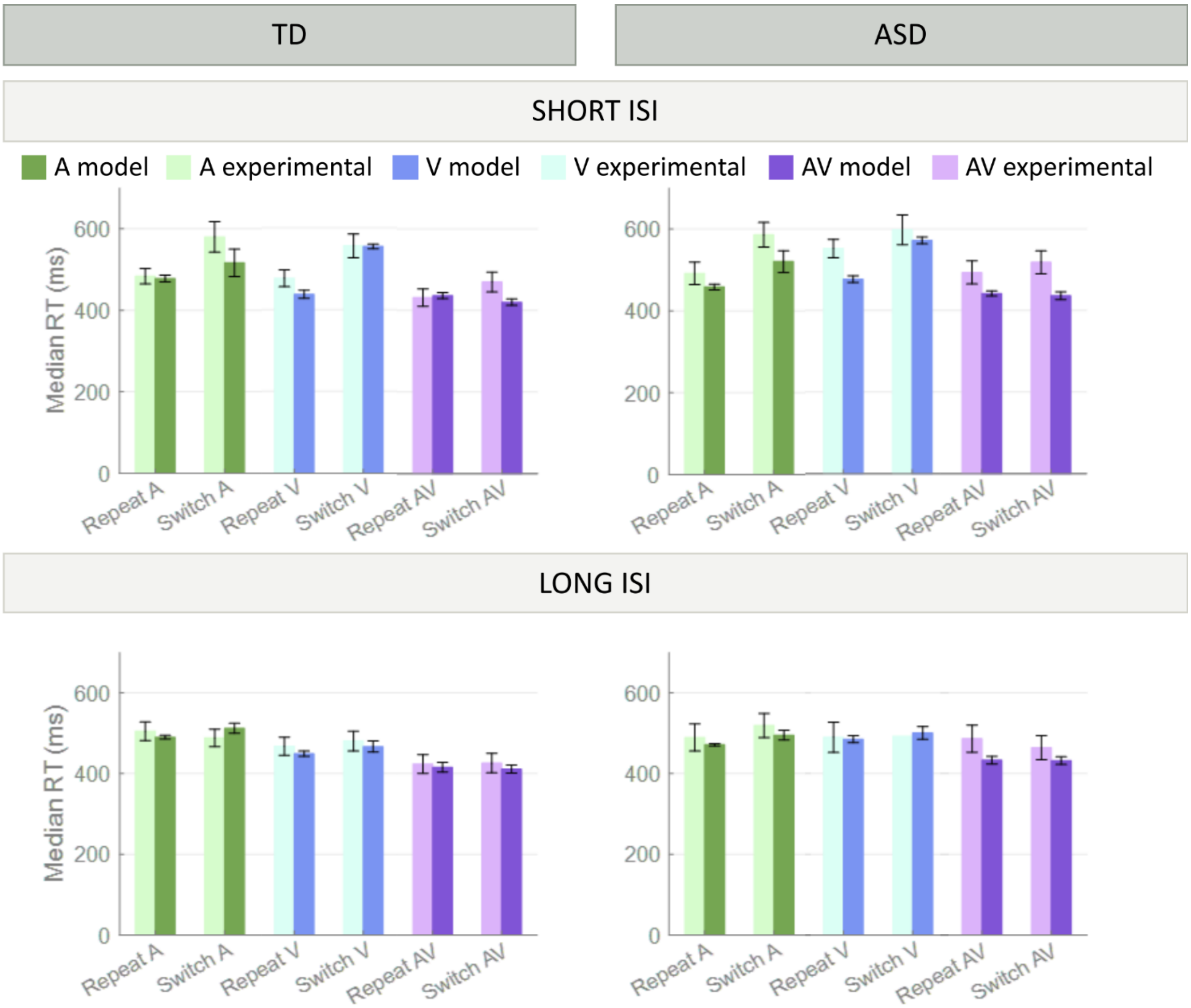

Figure S1 –Effect of ISI manipulation in the Competitive model configuration*. Simulated RTs from the Competitive default configuration are shown for both TD and ASD populations, under Short ISI (1–1.5 s) and Long ISI (2.5–3 s) conditions. Simulated RTs are compared with the empirical ones collected from Crosse's and colleagues under the same experimental conditions. Error bars indicate the standard error of the mean. The model closely replicates the empirical patterns in both populations, supporting the validity of the Competitive configuration in capturing the behavioral effects of ISI manipulation.*

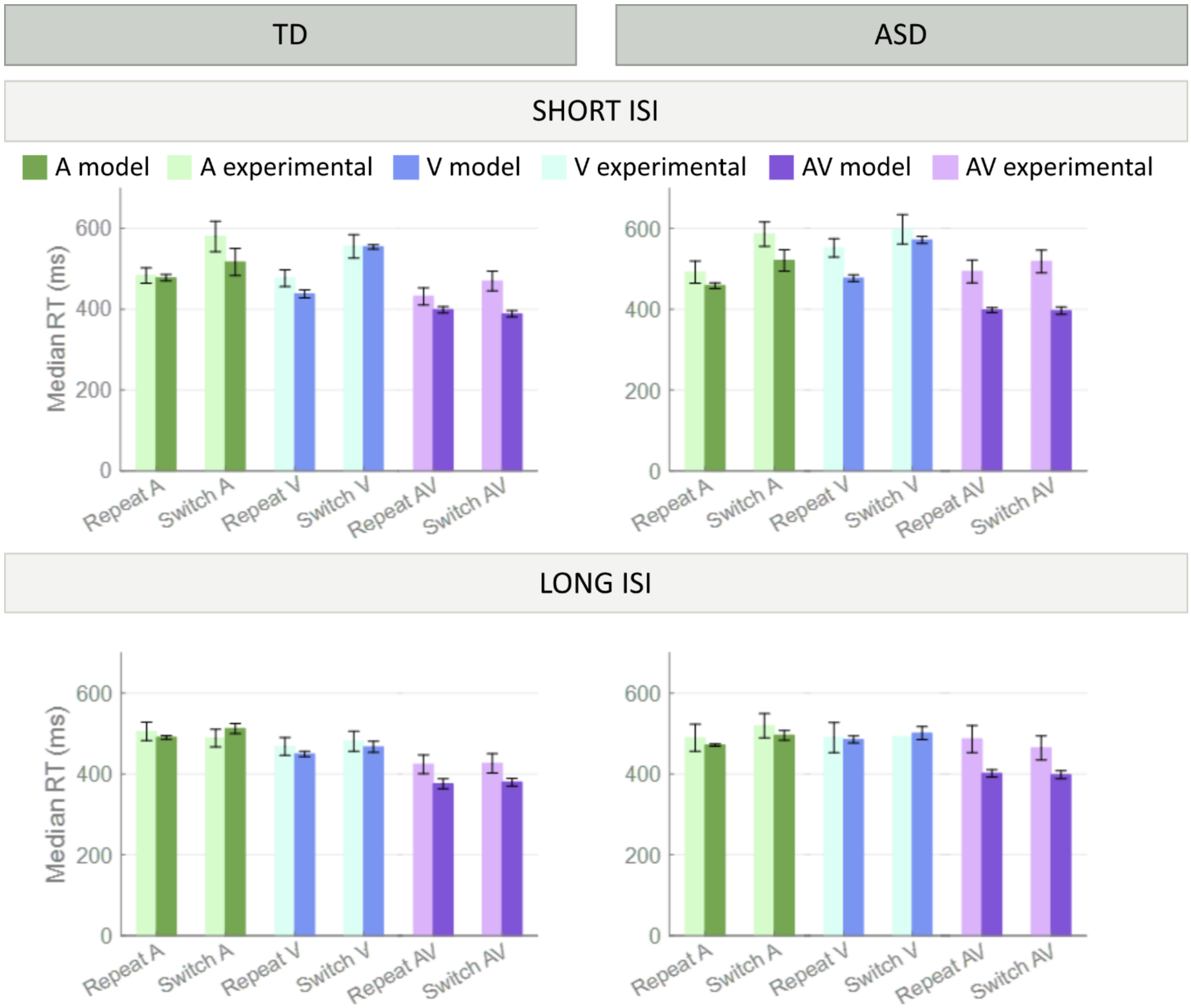

***Figure S2* – Long vs short ISI in the independent default configuration.** *Simulated RTs from the Independent default configuration are shown for both TD and ASD populations, under Short ISI (1–1.5 s) and Long ISI (2.5–3 s) conditions. Simulated RTs are compared with the empirical ones collected from Crosse's and colleagues under the same experimental conditions. Error bars indicate the standard error of the mean. Compared to the Competitive model, the Independent configuration provides a poorer fit to the empirical data in terms of ISI modulation.*

References

Ben-Yishai, R., Bar-Or, R. L., & Sompolinsky, H. (1995). Theory of orientation tuning in visual cortex. *Proceedings of the National Academy of Sciences*, *92*(9), 3844–3848. https://doi.org/10.1073/pnas.92.9.3844

Crosse, M. J., Foxe, J. J., Tarrit, K., Freedman, E. G., & Molholm, S. (2022). Resolution of impaired multisensory processing in autism and the cost of switching sensory modality. *Communications Biology*, *5*(1), 601. https://doi.org/10.1038/s42003-022-03519-1

Cuppini, C., Magosso, E., Bolognini, N., Vallar, G., & Ursino, M. (2014). A neurocomputational analysis of the sound-induced flash illusion. *NeuroImage*, *92*, 248–266. https://doi.org/10.1016/j.neuroimage.2014.02.001

Cuppini, C., Ursino, M., Magosso, E., Crosse, M. J., Foxe, J. J., & Molholm, S. (2020). Cross-sensory inhibition or unisensory facilitation: A potential neural architecture of modality switch effects. *Journal of Mathematical Psychology*, *99*, 102438. https://doi.org/10.1016/j.jmp.2020.102438

Jansen, B. H., & Rit, V. G. (1995). Electroencephalogram and visual evoked potential generation in a mathematical model of coupled cortical columns. *Biological Cybernetics*, *73*(4), 357–366. https://doi.org/10.1007/BF00199471

Maunsell, J. H., & Gibson, J. R. (1992). Visual response latencies in striate cortex of the macaque monkey. *Journal of Neurophysiology*, *68*(4), 1332–1344. https://doi.org/10.1152/jn.1992.68.4.1332

Molholm, S., Murphy, J. W., Bates, J., Ridgway, E. M., & Foxe, J. J. (2020). Multisensory Audiovisual Processing in Children With a Sensory Processing Disorder (I): Behavioral and Electrophysiological Indices Under Speeded Response Conditions. *Frontiers in Integrative Neuroscience*, *14*. https://doi.org/10.3389/fnint.2020.00004

Raij, T., Ahveninen, J., Lin, F., Witzel, T., Jääskeläinen, I. P., Letham, B., Israeli, E., Sahyoun, C., Vasios, C., Stufflebeam, S., Hämäläinen, M., & Belliveau, J. W. (2010). Onset timing of cross-sensory activations and multisensory interactions in auditory and visual sensory cortices. *European Journal of Neuroscience*, *31*(10), 1772–1782. https://doi.org/10.1111/j.1460-9568.2010.07213.x

Recanzone, G. H., Guard, D. C., & Phan, M. L. (2000). Frequency and Intensity Response Properties of Single Neurons in the Auditory Cortex of the Behaving Macaque Monkey. *Journal of Neurophysiology*, *83*(4), 2315–2331. https://doi.org/10.1152/jn.2000.83.4.2315

Treves, A. (1993). Mean-field analysis of neuronal spike dynamics. *Network: Computation in Neural Systems*, *4*(3), 259–284. https://doi.org/10.1088/0954-898X/4/3/002

Wendling, F., Bartolomei, F., Bellanger, J. J., & Chauvel, P. (2002). Epileptic fast activity can be explained by a model of impaired GABAergic dendritic inhibition. *European Journal of Neuroscience*, *15*(9), 1499–1508. https://doi.org/10.1046/j.1460-9568.2002.01985.x
